# Infection of human monocyte-derived dendritic cells by ANDES Hantavirus enhances pro-inflammatory state, the secretion of active MMP-9 and indirectly enhances endothelial permeability

**DOI:** 10.1186/1743-422X-8-223

**Published:** 2011-05-13

**Authors:** Delphine Marsac, Stephanie García, Alexandra Fournet, Adam Aguirre, Karla Pino, Marcela Ferres, Alexis M Kalergis, Marcelo Lopez-Lastra, Francisco Veas

**Affiliations:** 1 Pontificia Universidad Católica de Chile, Millennium Institute on Immunology and Immunotherapy, Laboratorio de Virología Molecular, Facultad de Medicina, Santiago, Chile; 2 UMR-MD3-University Montpellier 1, Comparative Molecular Immuno-Physiopathology Lab, Faculté de Pharmacie, 34093 Montpellier, France; 3 Universidad de Chile, Laboratorio de Comunicaciones Celulares. Centro de Estudios Moleculares de la Célula, Facultad de Medicina, Santiago, Chile; 4 Pontificia Universidad Católica de Chile, Laboratorio de Infectologia, Centro de Investigaciones Medicas, Facultad de Medicina, Santiago, Chile; 5 Pontificia Universidad Católica de Chile, Millennium Institute on Immunology and Immunotherapy. Departamento de Genética Molecular y Microbiología, Facultad de Ciencias Biológicas, Santiago, Chile

## Abstract

**Background:**

Andes virus (ANDV), a rodent-borne Hantavirus, is the major etiological agent of Hantavirus cardiopulmonary syndrome (HCPS) in South America, which is mainly characterized by a vascular leakage with high rate of fatal outcomes for infected patients. Currently, neither specific therapy nor vaccines are available against this pathogen. ANDV infects both dendritic and epithelial cells, but in despite that the severity of the disease directly correlates with the viral RNA load, considerable evidence suggests that immune mechanisms rather than direct viral cytopathology are responsible for plasma leakage in HCPS. Here, we assessed the possible effect of soluble factors, induced in viral-activated DCs, on endothelial permeability. Activated immune cells, including DC, secrete gelatinolytic matrix metalloproteases (gMMP-2 and -9) that modulate the vascular permeability for their trafficking.

**Methods:**

A clinical ANDES isolate was used to infect DC derived from primary PBMC. Maturation and pro-inflammatory phenotypes of ANDES-infected DC were assessed by studying the expression of receptors, cytokines and active gMMP-9, as well as some of their functional status. The ANDES-infected DC supernatants were assessed for their capacity to enhance a monolayer endothelial permeability using primary human vascular endothelial cells (HUVEC).

**Results:**

Here, we show that *in vitro *primary DCs infected by a clinical isolate of ANDV shed virus RNA and proteins, suggesting a competent viral replication in these cells. Moreover, this infection induces an enhanced expression of soluble pro-inflammatory factors, including TNF-α and the active gMMP-9, as well as a decreased expression of anti-inflammatory cytokines, such as IL-10 and TGF-β. These viral activated cells are less sensitive to apoptosis. Moreover, supernatants from ANDV-infected DCs were able to indirectly enhance the permeability of a monolayer of primary HUVEC.

**Conclusions:**

Primary human DCs, that are primarily targeted by hantaviruses can productively be infected by ANDV and subsequently induce direct effects favoring a proinflammatory phenotype of infected DCs. Finally, based on our observations, we hypothesize that soluble factors secreted in ANDV-infected DC supernatants, importantly contribute to the endothelial permeability enhancement that characterize the HCPS.

## Background

Hantaviruses are rodent-born enveloped RNA-viruses belonging to *Bunyaviridae *family. Two major severe pathologies associated to Hantaviruses have been reported: hemorrhagic fever with renal syndrome (HFRS) in the Eurasia and Hantavirus cardiopulmonary syndrome (HCPS) in the Americas. HCPS is more frequently associated (40%) to fatal outcomes than HFRS (<1%) [1]. Andes Hantavirus (ANDV) is the major etiological agent of the HCPS in South America, syndrome characterized by the presence of high amounts of pulmonary fluids leading to an edema evolving to a cardiogenic shock that synergistically acts with hypovolemia due to capillary leakage resulting in an abrupt cardiopulmonary collapse [2]. Although disease severity directly correlates with the viral RNA load [3], considerable evidence exists suggesting that immune mechanisms rather than direct viral cytopathology are indeed responsible for the massive vascular dysfunction and plasma leakage of HFRS and HCPS [4,5].

The hemorrhagic viruses, including the members of the *Bunyaviridae *as well as dengue viruses, target endothelial cells and immune cells, mainly monocyte-derived cells such as the professional antigen-presenting cells, Dendritic cells (DCs) [6-8]. DCs activation triggers their maturation and trans-endothelial migration occurring during wound healing or inflammation. These processes require extracellular matrix remodeling and involve changes in endothelial permeability regulated by the production of matrix metalloproteases (gMMPs) or vascular endothelial growth factor (VEGF). However, in excess, these soluble factors can have deleterious effects on endothelial cell integrity. Data from different reports show that endothelial cells infected by dengue virus trigger secretion of soluble factors such as VEGF and the decrease of VEGF-R2 receptor [9,10]. We have recently reported *in vitro *and *in vivo *showing that soluble factors secreted from DV-infected DCs enhance endothelial permeability and down-regulate expression of endothelial junction proteins, Pecam-1 and VE-cadherin in a gMMP-9-dependent manner [11]. More recently, complementary and convergent studies, to our own previous data on dengue, have reported that Hantavirus-infected endothelial cells enhances the permeability *via *the reduction of VE-cadherin expression due to its dissociation with VEGF-receptor2 (VEGF-R2) which, in turn, become associated with VEGF [12,13]. An accurate understanding of Hantavirus pathogenesis is pivotal to design *de novo *therapeutic or vaccine approaches that are still lacking against this hemorrhagic viral infection. In this study, we show that ANDV-infected DC are quickly activated and rapidly progress to an intermediate maturation and pro-inflammatory state that contributes to the increase of soluble factors in their supernatant able to trigger the enhancement of endothelial permeability.

## Methods

### Virus and cells

The primary isolate, ANDV strain CHI-7913 was propagated in the epithelial Vero-E6 cell line (ATCC CRL 1586). Titrated supernatants of these cells were used to infect, at a MOI of 1 for 2 h, human iDCs derived from peripheral blood monocytes (PBMC), as previously described [14]. In these experiments, UV (λ: 250 nm; 15 min)-irradiated ANDV was used as the negative control. Four days post-DC infection, ANDV N-protein was detected by indirect immunofluorescence (IFA) using a well characterized anti-ANDV N monoclonal antibody (MAb) [15]. Total RNA was extracted using the High Pure viral nucleic acid kit (Roche Molecular Biochemicals, Mannheim, Germany) following the manufacture's protocol and 1 μl of total RNA was amplified in a one step RT-PCR (SuperScript III One-Step RT-PCR with Platinum Taq, Invitrogen) using primers that recognize the nucleocapsid coding region (forward primer: 5' ACA CGA ACA ACA GCT CGT GAC '3 and reverse primer: 5' AGG CTC AAG CCC TGT TGG ATC '3). To assess the viral infectivity, from ANDV-positive DCs, their supernatants were used to infect Vero-E6 cells.

### Phenotype profiling of ANDES-infected DC

The influence of ANDV (3 h post-viral) infection of iDCs (as described above) was assessed through the expression of key cell surface proteins CD80, CD83, CD86 and HLA-DR, known to be associated with the mature DCs (mDCs) phenotype. Pro-inflammatory profile of ANDV-infected iDCs was assessed through the expression level of some pro and anti-inflammatory cytokines as well as gMMP. As infection of iDCs by other Hantaviruses induces the pro-inflammatory cytokines expression [7]. Lipopolysaccharide (LPS)-pulsed iDCs were used as a positive control for iDCs maturation, while iDCs incubated with fresh culture medium (mock) were used as a negative control. Supernatants of Mock-DCs, ANDV-infected DCs, and LPS-pulsed DCs were assessed for the production of the pro-inflammatory cytokine, TNF-α, (ELISA kit OptEIA™ Human TNF ELISA Kit II; Pharmingen, Franklin Lakes, NJ).

### Functional assessment of ANDV-infected DC

The effect of ANDV infection on the endocytic capacity of DCs was assessed by measuring the FITC-conjugated Dextran up-take at 37°C [16]. In this assay, 18 h LPS-pulsed iDCs were used as a control for cell maturation assessment, while iDCs, incubated at 4°C, were used as a control for the immature stage with lowest capacities of endocytosis. The apoptotic level of ANDV-infected DCs was assessed by flow cytometry using the Annexin V assay [17] using the same previous controls.

### Gelatinolytic MMP-9 secretion

Intra-cellular expression of gMMP-9 in ANDV-infected DCs (3 h-post infection) was characterized by Western blotting, using a mouse anti-gMMP-9 MAb (MAB13416; Chemicon International, Temecula, CA). The gelatinolytic activity of gMMP-9 DC supernatants (SN) was assessed by gelatin zymography [18]. In these experiments fresh medium (SN-Mock) was used as a negative control for maturation, while medium containing LPS (1 μg/ml) (SN-LPS) was used as a positive control.

### Endothelial monolayer permeability assessment

The capacity to modify the vascular permeability of ANDV-infected DC supernatants was measured *in vitro*, using a monolayer of primary human umbilical vascular endothelial cells (HUVEC) [19] Briefly, HUVEC obtained from pooled donors were plated onto collagen-coated transwell inserts (0.4 μm-pore, 6.5 mm-diameter Transwell-COL; Costar) and once in confluence cells, were exposed for 18 h to mock-SN, virus- free SN from ANDV infected DCs, or to TNF-α (50 nM). Monolayer endothelial permeability was assayed with the standard cell permeability assay according to the Chemicon ECM-640 procedure (Millipore), by adding FITC-conjugated dextran to the upper chamber of the transwell inserts and evaluating the migration of FITC-conjugated Dextran to the lower chamber by using a TECAN fluorometer at 490/530 nm. In this transwell system, endothelial monolayer permeability is directly proportional to the flux of 70-kDa dextran passing through the HUVEC monolayer.

### Statistical Methods

Data sets were compared using Kruskal & Wallis analysis of variance was performed with the GraphPad Prism 4.03 software. Two-sided p-values were considered.

## Results and Discussion

**Figure 1:**
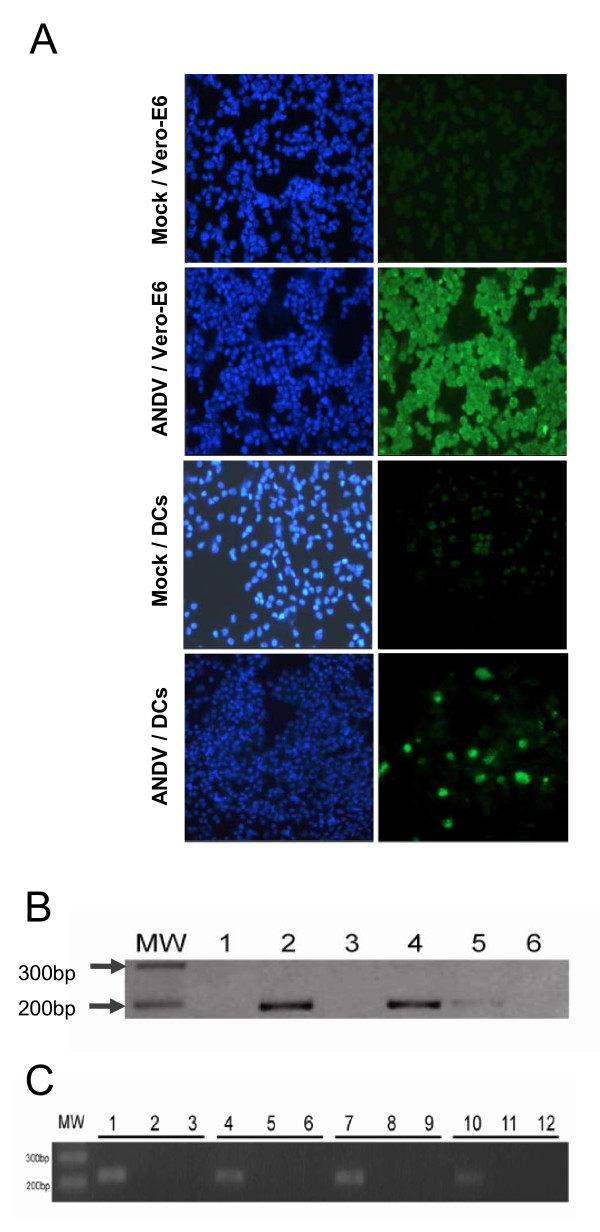
**Detection of ANDV infection of primary human dendritic cells**. **(A) **Vero E6 epithelial cells and human immature DCs infected with ADNV (strain CHI-7913). ANDV-nucleocapsid (N) protein detected by IFA by incubating ANDV-infected with a mouse anti-ANDV N MAb revealed by an FITC-conjugated anti-mouse IgG Ab (Green, right column) while the corresponding cell nuclei were stained with 4',6-diamidino-2-phenylindole (DAPI, blue, left column). Negative control (mock) cells were incubated with supernatant from uninfected Vero-E6 cells. **(B) **Total RNA was extracted from uninfected LPS-Pulsed DCs (lane 1), ANDV-infected LPS-pulsed DCs (lane 2), uninfected iDCs (lane 3), ANDV-infected iDCs (lane 4), ANDV-infected Vero-E6 cells (lane 5), and used as template in a RT-PCR reaction designed to specifically amplify the viral S RNA. This assay also included a negative RT-PCR control (lane 6). MW is a molecular weight marker (1 Kb, Fermentas, Burlington, Canada). **(C) **DCs generated from primary monocytes, recovered from four healthy donors, were incubated with ANDV (lanes 1, 4, 7, 10), UV-irradiated ANDV (lanes 2, 5, 8, 11) or pulsed with LPS (lanes 3, 6, 9, 12). Total RNA was extracted from cell supernatants and used as a template in a RT-PCR reaction designed to specifically amplify the viral S RNA. MW is a molecular weight marker (100 pb, Fermentas).

Primary isolate, ANDV strain CHI-7913, was shown to efficiently propagated in the epithelial Vero-E6 cell line (Figure 1A), as previously reported [20], and that their titrated supernatants efficiently targeted and infected human iDCs (Figure 1A). At 4 days post-infection ANDV N-protein was detected by IFA using an anti-ANDV N MAb. Consistent with the cell immunofluorescence data, the presence of ANDV RNA in extensively washed infected DCs was confirmed by an ANDV-specific RT-PCR [21] (Figure 1B). The presence of both, viral antigens and RNA, in DCs does not necessarily imply viral replication, as the observations can be explained by the uptake of exogenous viral particles by cells without productive infection. To check for this option, infected DCs were extensively washed and fresh medium was added. Cell supernatants were recovered, total RNA isolated as described above and subjected to an ANDV-specific RT-PCR. The presence of viral RNA in DC supernatants was confirmed (Figure 1C), suggesting shedding of *de novo *generated viral particles. To assess the viral infection capacity, supernatants from ANDV-positive DCs were used to infect Vero-E6 cells. Five days post-infection, the presence of ANDV in Vero-E6 cells was confirmed by both IFA and RT-PCR as described above (data not shown). Together these observations strongly suggest that, consistent with other members of the Hantavirus genus of the *Bunyaviridae*, ANDV is able to infect and replicate in human DCs. Consequently, we explored the possible effects of this viral infection on both DC phenotypes and functionalities.

**Figure 2:**
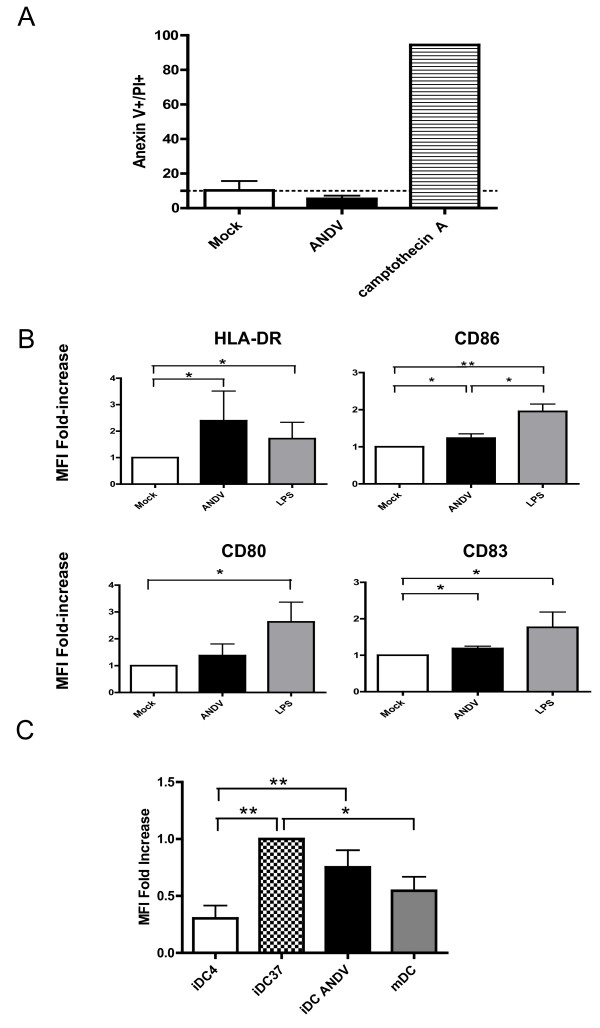
**Impact of ANDES virus infection on iDC phenotype and functions**. (**A**) The impact of ANDV infection on DCs viability was detected by using the Annexin V-propidium iodide (PI) method. In these assays, DCs treated with camptothecin A (4 μM for 18 h) were used as a positive apoptosis control. **(B) **DCs surface markers; CD80, CD86, CD83 and HLA-DR were analyzed in ANDV-infected iDCs by flow cytometry four days post-ANDV infection, while LPS-pulsed DCs and uninfected iDCs (mock) were used as controls. Bar graphs represent the fold-increase expression of these surface markers as compared their expression in mock control. Data are means of three independent experiments: *, *p*< 0.05; **, *p *< 0.01. **(C) **The endocytic capacity of ANDES infected iDCs, LPS-matured DCs (mDC), uninfected iDCs incubated at 37°C (iDC 37), and uninfected iDCs incubated at 4°C (iDC 4) was assessed using a FITC-conjugated Dextran (30 μg). Endocytosis was analyzed by flow cytometry after 2 h of incubation. Mean fluorescence intensity values within the gate for the different endocytically active stages were plotted. Bar graphs show the fold-increases of the mean fluorescence intensities (MFI), relative to the mock control (iDCs 37). For each experiment, 10 000 gated cells were evaluated. Data are means of five independent experiments. *, *p*< 0.05; **, *p *< 0.01.

The impact of DC infection by ADNV on cell viability, maturation and endocytosis was assessed. The apoptotic level, evaluated by flow cytometry using the Annexin V assay [17], showed that infection of iDCs with ANDV does not affect cell viability (Figure 2A), observations in line with what has been reported for other hantaviruses [6,22]. In addition, ANDV infection did not induce any detectable cytopathic effect (data not shown). DCs play a pivotal role as antigen-presenting cells in the antiviral immune response. It is known that infection of iDCs by diverse viruses stimulates cell homing to inflammatory sites as well as their maturation into antigen-presenting cells (APC), a process essential for the initiation and modulation of T cell-mediated immune responses. Hence, we next evaluated whether ANDV infection of iDCs had a direct effect on the expression of key cell surface proteins CD80, CD83, CD86 and HLA-DR, known to be associated with a mature DCs (mDCs) phenotype. Immature DCs were infected with ANDV as described above. ANDV infection of iDCs induced a significant increase of HLA-DR, a marker protein that plays a pivotal role in guiding the development and activation of CD4^+ ^T helper cells. Markers CD83 and CD86 also increased, albeit to a much lower extent than in LPS-pulsed iDCs conditions. As expected, expression of all surface markers increased in LPS-pulsed iDCs as compared to mock-DCs (Figure 2B). These data suggest that ANDV infection of human DCs induced cell maturation, and that ANDV infection alters the expression of HLA-DR on DCs. Constitutive expression of HLA-DR molecules is associated to professional antigens presenting cells (APCs) such as DCs, this basal expression can be enhanced in an environment enriched with proinflammatory cytokines [7,23].

Immature DCs efficiently capture and process antigen, a characteristic cell function that decreases with maturation [7,8,24-27]. Therefore, we next sought to establish whether the partial DC maturation phenotype (Figure 2B) was correlated with its decreased ability to capture antigens. To this purpose, we studied the effect of ANDV infection on the endocytic capacity of DCs by measuring the up-take of FITC-conjugated Dextran at 37°C [16]. Results indicate that ANDV-infected DC exhibited a reduced endocytic capacity as compared to uninfected iDCs (Figure 2C). However, in our conditions, infected DCs exhibited a higher endocytic activity than the used dose of LPS to pulse iDCs (positive control). Together, these observations suggest that ANDV infection does indeed reduce antigen capture by DCs, supporting a role of ANDV infection in cell maturation.

**Figure 3:**
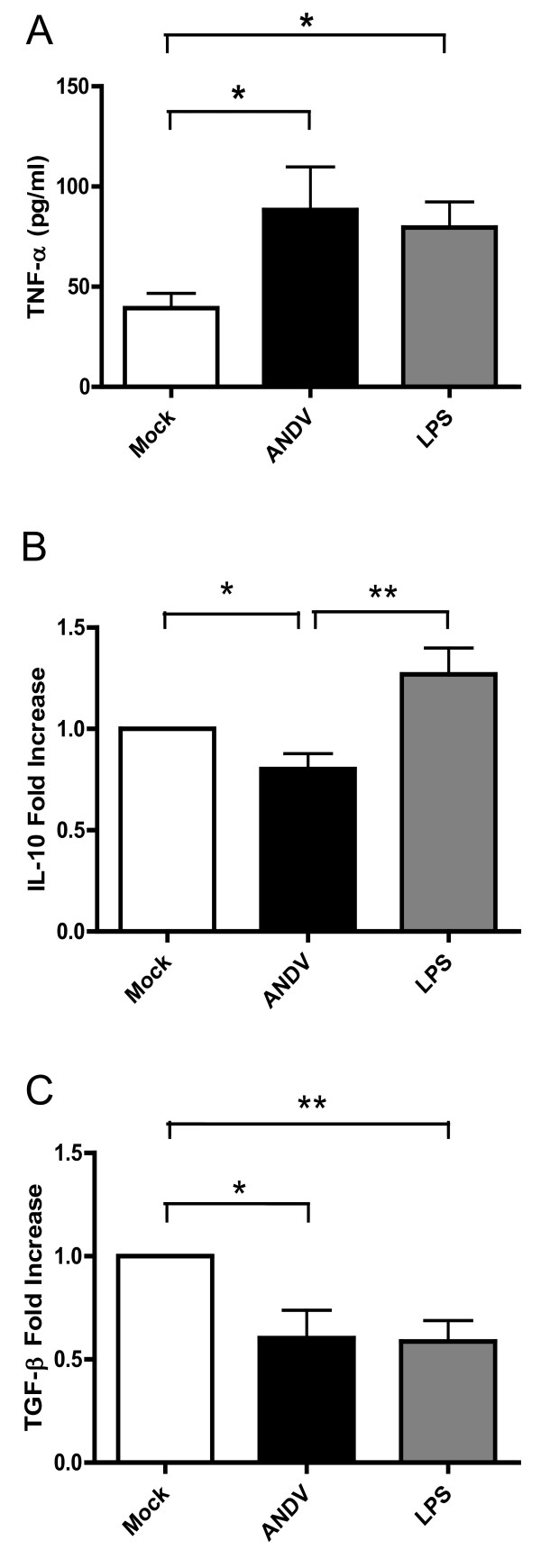
**Inflammatory state of ANDV-infected iDCs**. The level of TNF-α **(A) **IL-10 **(B)**, and TGF-β **(C) **in supernatants of uninfected DCs (Mock), ANDV-infected iDCs (MOI = 1) (ANDV), or LPS-pulsed DCs, was assessed by ELISA respectively at 3 h (A) and 48 h (B and C) post-ANDV infection. In (B) and (C), bar graphs depict the fold-increase of cytokine production relatively to mock. Data are means of five to eight independent experiments *, *p*< 0.05; **, *p *< 0.01.

The presence of high levels of pro-inflammatory cytokines both in plasma and lungs have been reported as pathological markers associated with HCPS in humans. These excesses of pro-inflammatory cytokines are secreted in patients by both hantavirus-activated macrophages and specific T cells [28]. Particularly, the levels of the pro-inflammatory cytokine TNF-α in HCPS patient sera are the dramatically elevated [29,30]. Infection of iDCs by other Hantaviruses induces the production of pro-inflammatory cytokines as well [7]. Therefore, we sought to evaluate whether ANDV infection induces a similar DC phenotype. Supernatants of Mock-DCs, ANDV-infected DCs (3 h post-viral infection), and LPS-pulsed DCs were assessed for the production of the pro-inflammatory cytokine, TNF-α. In agreement with what has been described for other Hantaviruses, supernatants harvested from ANDV-infected DCs display a significant increase in TNF-α as compared with supernatants from uninfected iDCs (Figure 3A). In a similar experimental setting, we also evaluated the expression of IL-10 and TGF-β, two anti-inflammatory cytokines [31]. Interestingly, in supernatants harvested from ANDV-infected DCs, IL-10 levels were weakly altered as compared with uninfected DCs, while TGF-β levels decrease was more pronounced (Figure 3B and 3C). Thus, our observations suggest that ANDV-infected DCs exhibit a pro-inflammatory cytokine profile.

**Figure 4:**
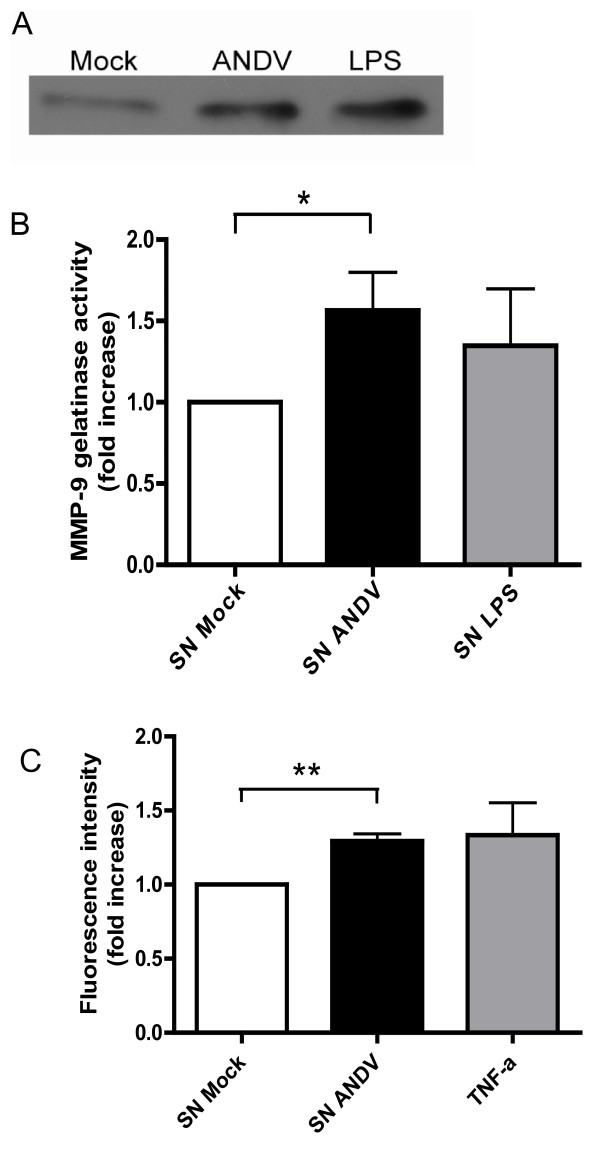
**Secretion of active gMMP-9 and endothelial permeability enhancement are triggered by ANDV infection of iDCs**. (**A**) The gMMP-9 expression in ANDV-infected iDCs was assessed by Western blotting. Uninfected iDCs (Mock), ANDV-infected iDCs (ANDV) and LPS-treated iDCs (LPS) were collected 3 h post-infection. Forty micrograms of total proteins from cell lysates were separated in an 8% SDS-PAGE gel. Following an electrical protein transfer onto a nitrocellulose membrane, an anti-gMMP-9 antibody was used to probe the presence of gMMP-9 that was revealed using an HRP-conjugated anti-mouse IgG Ab. (**B**) Supernatants from ANDV-infected DCs or Mock cells collected 3 h post-infection. Gelatinase activity was assayed by zymography. Gelatinolytic activity was quantified by gel densitometry using the Image J Software. Data are presented as the fold-increase of gMMP-9 activity in supernatants. Statistical significance (**, *p *< 0.01) was determined from five independent experiments. (**C**) Enhancement of the endothelial cells permeability induced by supernatant from ANDV-infected iDCs. HUVEC confluent monolayers plated onto collagen-coated transwell inserts were incubated with either ANDV supernatant, mock control (uninfected DCs supernatant) or with TNF-α (50 ng/ml) as positive control. Following 18 h at 37°C in CO2 5% after addition, within the top chamber, of 500 μg/ml FITC-conjugated Dextran, paracellular permeability was measured by reading in the bottom chamber containing the infiltrated FITC-dextran at an excitation wavelength of 485 nm and an emission of 530 nm. Data represent means of five independent experiments. **, p < 0.01.

Upon antigen capture, DCs undergo a process of maturation and a lymph nodes homing, where they activate the adaptive immune system. The process of DC trafficking is complex. This phenomenon requires the enhancement of the vascular permeability, which could involve the expression of several components, such as cell to cell junction proteins including VE-cadherin, PECAM-1, occludin, claudins, as well as soluble factors, including, IL-1β, TNF-α, VEGF, and Kinins [32-34]. The gelatinolytic matrix metalloproteases (gMMP)-2 and 9 are reported as one of the major actors of this crucial phenomenon during fetal development and wound healing [35]. Interestingly, factors such as the inflammatory cytokines IL-8, TNF-α and IL-1β as well as VEGF [36] are in the upstream of the gMMP expression pathway in human monocytes [37]. Additionally, even if iDC produces gMMP, DC increase the MMP-9 production along DC maturation progression [38]. Interestingly, certain viruses can play opposite roles in DC maturation, for example the human Cytomegalovirus (hCMV) inhibits cell maturation to escape the immune system (4), whereas some others, such as HIV-1 [18], Dengue virus [11], and West Nile virus [39], enhance DC maturation, the secretion of active gMMP-9, as well as plasma vascular leakage. Thus, to extend our previous observation on the fact that DCs have a proinflammatory profile that can be compatible with the secretion of gMMP-9, we analyzed the expression and activity of gMMP-9 in cells and its presence in DC supernatants (SN). Selection seemed adequate as the levels of IL-10, which has been reported to inhibit MMP-9 induction, is weakly altered by ADNV infection (Figure 3B) and DC maturation is known to increase the level of active MMP-9 secretion. Intra-cellular expression of gMMP-9 in ANDV-infected DCs (3 h-post infection) was characterized by Western blotting (Figure 4A), using a mouse an anti-gMMP-9 MAb. Data show that gMMP-9 expression is indeed elevated in ANDV-infected DCs as compared with mock iDCs (Figure 4A). Gelatinolytic MMP-9 is secreted as a proenzyme, which remains inactive unless it is activated by the removal of the propeptide domain by proteolytic enzymes. The gelatinolytic activity of gMMP-9 was therefore assessed in cell supernatants (SN) by gelatin zymography [18]. Strikingly, ANDV-infected DC supernatants exhibited an elevated gMMP-9 activity as compared with SN-mock (Figure 4B). Furthermore, gMMP-9 activity was similar to that obtained by the LPS-induction of DC maturation (SN-LPS; Figure 4B).

ANDV belongs to the group of hemorrhagic fever viruses known for inducing different forms of vascular leakage in different organs. For some of these hemorrhagic fever or encephalitic viruses the mechanism associated to their capacity to enhance endothelial permeability *in vitro *and *in vivo *has been reported [11,18,39]. Upon viral infection, the vascular permeability phenomenon is principally due to viral-dependent dysfunctions of endothelial cells junctions that can be directly induced by the action of factors from viral activated endothelial cells, such as VEGF or indirectly induced by factors from immune viral-activated cells, *via *the MAP-kinase pathway [11,18]. For these reasons, we wonder if ANDV infection induced the production of soluble factors that could indirectly enhance endothelial permeability. To evaluate this possibility, the capacity of ANDV infected DCs-supernatants to modify the permeability was measured through the amounts of FITC-conjugated dextran that passed thru a monolayer of primary human umbilical vascular endothelial cells (HUVEC) seeded onto transwell plates that considered as vascular model [19]. Following the exposure of confluent HUVEC to SN from ANDV infected DCs, or to controls, the amounts of FITC-conjugated dextran detected in the lower chamber of the transwell system (Figure 4C) suggest that cell supernatant from ANDV-infected DCs contains soluble and active factors favoring the increase of the HUVEC monolayer permeability.

Altogether, the *in vitro *data presented herein show that ANDV infects and replicates in DCs, inducing a partial maturation and pro-inflammatory phenotype, while increasing the expression and secretion of active gMMP-9 as well as that supernatants from ANDV-infected DCs were able to enhance the permeability of a monolayer of primary human vascular endothelial cells.

## Conclusion

The herein presented data and data recently published in the literature are not only convergent but also complementary, they allow to speculate that both indirect effects of ANDV on DCs and direct effects of pathogenic hantaviruses, such as ANDV, on endothelial cells generate conjugated inflammatory consequences that result in the increased endothelial permeability associated to this virus infection. A better understanding of major mechanisms involved in vascular pathogenesis observed in HCPS is pivotal to progress in the design of therapies that today remain unavailable for this serious life-threatening hemorrhagic viral disease.

## Competing interests

The authors declare that they have no competing interests.

## Authors' contributions

DM carried out the BSL3 experiments, hantavirus cultivation, and most of the experiments described in this paper, manuscript draft; SG participated to all of those experiments; AF zymograms and experimental permeability studies set-up. AA performed the statistical analysis. KP RT-PCR; MF BSL-3 facilities; AK DCs PBMC-derived and draft revision; MLL data analysis and manuscript revision; FV designed the project, data interpretation, manuscript revisions, finalized and approved the manuscript. All authors read and approved the final manuscript.

## Authors' informations

DM was a FONDECYT post-doctorate fellowship; SG is PhD student and KP is a technician at the MLL lab; AF is from the technical staff of the FV lab.
